# Supplementary material for: Transcriptomic Insights into Mechanisms of Early Seed Maturation in the Garden Pea (Pisum sativum L.)
Source: Cells. 2020 Mar 23;9(3):779. doi: 10.3390/cells9030779 (PMC7140803; doi:10.3390/cells9030779)

# Zhewan−1(10) vs Zhon

## Global Summary

%DE = 0.84

# genes with fdr < 0.2  = 1681  ( 655 + / 1026  −)

# genes with fdr < 0.1  = 1569  ( 593 + / 976  −)

# genes with fdr < 0.05  = 1392  ( 506 + / 886  −)

# genes with fdr < 0.01 = 1141  ( 383 + / 758  −)

# genes in genesets = 0

<FC> = 0

<t−score> = −6127969.07

<p−value> = 0

<fdr> = 0.16

**Portrait**

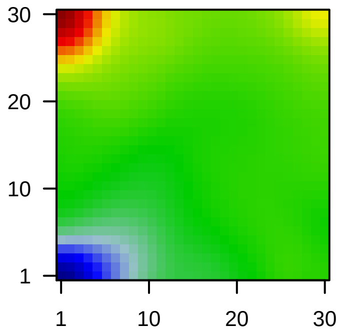


**Top 100 DE genes**

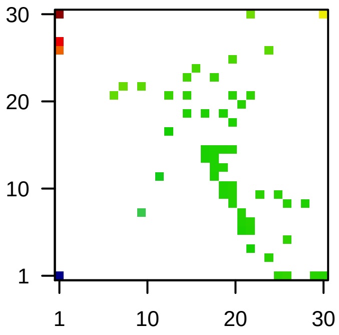


## Global Genelist

| Rank | ID | log(FC) | p−value | fdr | Metagene | Description |
|---|---|---|---|---|---|---|
| 1 | TRINITY_DN4 | −5.56 | 0e+00 | 3e−10 | 1 x 1 | |
| 2 | TRINITY_DN5 | 0.02 | 1e−12 | 4e−07 | 15 x 23 | |
| 3 | TRINITY_DN1 | 0 | 5e−09 | 4e−07 | 20 x 10 | |
| 4 | TRINITY_DN1 | 0 | 5e−09 | 4e−07 | 17 x 14 | |
| 5 | TRINITY_DN1 | 0 | 6e−09 | 4e−07 | 17 x 14 | |
| 6 | TRINITY_DN7 | 0 | 6e−09 | 4e−07 | 24 x 3 | |
| 7 | TRINITY_DN1 | 0 | 7e−09 | 4e−07 | 17 x 14 | |
| 8 | TRINITY_DN5 | 0 | 7e−09 | 4e−07 | 17 x 14 | |
| 9 | TRINITY_DN7 | 0 | 9e−09 | 4e−07 | 18 x 13 | |
| 10 | TRINITY_DN1 | 0 | 9e−09 | 4e−07 | 18 x 14 | |
| 11 | TRINITY_DN7 | 0 | 1e−08 | 4e−07 | 21 x 6 | |
| 12 | TRINITY_DN7 | 0 | 1e−08 | 4e−07 | 18 x 13 | |
| 13 | TRINITY_DN2 | −0.12 | 1e−08 | 4e−07 | 10 x 8 | |
| 14 | TRINITY_DN1 | 0 | 1e−08 | 4e−07 | 26 x 1 | |
| 15 | TRINITY_DN4 | 0.11 | 2e−08 | 4e−07 | 10 x 22 | |
| 16 | TRINITY_DN3 | 0 | 2e−08 | 9e−07 | 17 x 14 | |
| 17 | TRINITY_DN7 | 0 | 2e−08 | 9e−07 | 18 x 13 | |
| 18 | TRINITY_DN3 | 0 | 3e−08 | 9e−07 | 17 x 15 | |
| 19 | TRINITY_DN1 | 0 | 3e−08 | 9e−07 | 22 x 7 | |
| 20 | TRINITY_DN4 | 0 | 3e−08 | 9e−07 | 26 x 9 | |

**p−values**

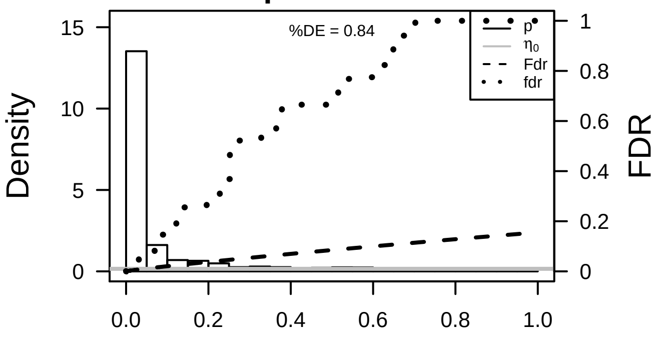

Supplement: Supplementary file 1 [file cells-09-00779-s001.zip › Supplementary materials/FileS4.zip!/FileS3/TFs - Results/Summary Sheets - Differences/Reports/Zhewan.1.10..vs.Zhongwan.6.10..pdf]
